# It is child’s play: Caregiver and playworker perspectives on a community park-based unstructured play program

**DOI:** 10.1371/journal.pone.0311293

**Published:** 2024-09-30

**Authors:** Gavin R. McCormack, Calli Naish, Jennie Petersen, Dalia Ghoneim, Patricia K. Doyle-Baker

**Affiliations:** 1 Department of Community Health Sciences, Cumming School of Medicine, University of Calgary, Calgary, Canada; 2 Institute for Public Health, University of Calgary, Calgary, Canada; 3 Libin Cardiovascular Institute, University of Calgary, Calgary, Canada; 4 Alberta Children’s Hospital Institute, University of Calgary, Calgary, Canada; 5 Faculty of Arts, Department of Communication, Media and Film, University of Calgary, Calgary, Canada; 6 Faculty of Kinesiology, University of Calgary, Calgary, Canada; La Trobe University - Melbourne Campus: La Trobe University, AUSTRALIA

## Abstract

Unstructured play is characterized as play that is child initiated and determined, allowing children the freedom and flexibility to engage in activities, including taking risks, without adult intervention. However, playworkers and caregivers are important mediators of children’s unstructured play. Studies have investigated playworker and caregiver perspectives regarding play, yet few have done so within the context of community based unstructured play interventions. Our qualitative study explored knowledge, attitudes and perspectives regarding play among playworkers (“play ambassadors”) and caregivers exposed to a community based unstructured play intervention implemented in Calgary, Canada. The intervention aimed to encourage unstructured and risky play in local parks via loose parts. Between August and October 2020, ten caregivers and four play ambassadors completed individual semi-structured interviews. Using thematic analysis, we identified four overarching themes. *The importance of play* (theme 1) reflected play ambassador and caregiver perspectives about how unstructured and risky play promoted health and development and offered opportunities for enjoyment, exploration, and social interaction. *Perceptions of the play environment* (theme 2) reflected play ambassador and caregiver experiences of the play hubs including the role of loose parts in shaping play. *Challenges and triumphs in promoting play* (theme 3) reflected play ambassador experiences in facilitating play as well as interactions between caregivers and play ambassadors. *Play hub impacts on the community* (theme 4) reflected play ambassador and caregiver perspectives on the role of the play hubs in facilitating social interactions and community engagement. Play Ambassadors and caregivers shared common beliefs about the benefits of unstructured and risky play and about the play hubs affects on facilitating social interactions. Community based unstructured play interventions may support play and promote a sense of community.

## Introduction

Unstructured play includes activities that are self-directed and intrinsically motivated and lack delineated rules [1,2]. In children, unstructured play can enhance physical literacy, physical, social, mental, and emotional health, cognitive development, risk management skills, and resiliency [3]. Unstructured play can incorporate activities that encourage risk-taking. Risky play offers children an opportunity to engage in self-directed activities that challenge their physical limits, but that also include risk for physical harm or injury [4,5]. Risky play can include activities involving great heights, high speed, dangerous tools, dangerous elements, rough-and-tumble play and opportunities that involve getting lost or disappearing, as well as loose parts and messy play [4–6]. However, opportunities for risky play are declining [1,7,8]. This decline has been attributed to children’s reduced access to unstructured play opportunities [9], stricter regulations governing the types of play activities that can be undertaken in structured or organized environments [10], caregiver prioritization of structured activities [11–13], and increased safety concerns regarding children’s outdoor play [10,14].

Caregivers often worry about injuries from their children’s risky play [4,5]. A study found 9% of European parents feared play-related injuries [15], while New Zealand parents allowed their children limited risky activities but felt overly restricted by safety regulations [6]. Despite concerns, most caregivers support risky play for developing risk management and promoting growth [16–20]. Similarly, play professionals (e.g., playworkers and educators) perceived benefits in promoting and encouraging unstructured and risky play [21,22]. Supportive play environments and supportive adults (e.g., caregivers and playworkers) may be needed to facilitate unstructured play and risk taking that balances child independence and self-direction with safety [23,24]. Perceptions of unstructured and risky play have been explored among caregivers [6,12,17,25–27] and play professionals [18,21,22,28–30] however, much of the evidence concerns play in institutional settings (e.g., schools and childcare facilities).

### Study rationale

Our study is part of a larger evaluation of public programming designed and delivered by a recreation facility (Vivo for Healthier Generations; ‘Vivo’) in Calgary (Alberta, Canada) aimed at providing physical activity opportunities to families through play. The Vivo Play Project offered different types of play opportunities including pop-up loose parts play events at local community parks, referred to as “play hubs” that were supervised by playworkers (“play ambassadors”) [31,32]. Play ambassadors were tasked with setting up the play hubs in parks, managing child sign-ins, removing hazards, motivating children, and conveying to parents the importance of unstructured and risky play. The play hubs were offered weekly throughout the year, including week and weekend days, between autumn 2019 and summer 2022, however they were suspended from March to September 2020 and December 2021 to February 2022 to comply with the COVID-19 pandemic public health measures. The play hubs aimed to offer an environment that promoted free play, during which the play ambassadors refrained from instructing children about how to play [33]. Play ambassadors underwent training, which included the completion of education modules covering theoretical and practical aspects of play work [34–37], risky play [4,38] and loose parts play [39].

The play hubs encouraged unstructured and risky play by providing children with loose parts in local parks. Loose parts play involved creating play spaces that provided access to a variety of materials including conventional (e.g., sports equipment and toys) and unconventional (e.g., natural elements, tools, fabrics, utensils) play items, that children used in self-determined ways [39–41]. Loose parts play fosters creativity and imagination through self-directed problem solving [41,42] and offers opportunities for risk taking as children engage with unfamiliar materials and challenging activities and environments [6,43,44]. The play hubs aligned with the recommendations and practical considerations outlined in Canadian Public Health Association’s “Loose Parts Policy” [45]. The play hub design and implementation have been described elsewhere [31].

There is a dearth of studies exploring caregiver and play professional perspectives of play associated with community based outdoor unstructured play, yet this evidence is vital in developing effective programs. The purpose of our study was to explore the knowledge, attitudes and perspectives regarding outdoor unstructured and risky play among caregivers and play ambassadors exposed to these play hubs. Our study sought to answer the following questions: 1) What are the perceptions and perspectives on play, unstructured play, and risky play among caregivers and playworkers exposed to a community-based loose parts intervention and do these two groups share similar or have differing perspectives on play, and; 2) what were the experiences of the play hubs from the perspective of caregivers whose children engaged in these events, and of the play ambassadors who delivered and facilitated these events?

## Materials and methods

### Conceptual framework and positioning

This study utilized a constructivist approach in exploring play ambassadors’ and caregivers’ ideas about children’s play, and their perceptions of a community-based loose-parts play initiative. This approach was valuable as it acknowledges that play ambassadors’ and caregivers’ perceptions are shaped by their unique and individual social lenses [46]. Furthermore, this approach recognizes the position of the researcher as contributing to the ideas and insights of the work and emphasizes the importance of conversation between researcher and research participant, and the dialogic nature of this type of qualitative research [46,47]. Additionally, this approach required the research team (GRM, CN, PKD-B, JAP, and DG) to consider how their own identities were important to the research process, as these identities influenced the interpretations of participant’s responses. The research team included middle-aged cis-gendered men and women, with undergraduate and graduate level educations, and with experience as coaches, child caregivers, or parents. Their expertise included backgrounds in sports science and public health, and they shared a collective interest in play, physical activity, and sport. Importantly, as this evaluation was not focused on the delivery of the play hubs, but the experience of the play hubs as reported by caregivers and play ambassadors, this researcher reflexivity was imperative to this study as children’s access to play, especially unstructured play is largely determined by adults such as parents and playworkers [9,12–14].

### Sampling and recruitment

We recruited caregivers by email invitation, via purposive sampling from those who had completed a cross-sectional survey (including 345 caregivers of at least one child 5–17 years of age) and who affirmed their interest in participating in future research [48]. The survey, launched in April 2020, was administered to a random sample of households (n = 1124) located within the geographical catchment area of Vivo’s recreational facility. Caregivers of children who had attended a play hub prior to the COVID-19 pandemic were eligible to participate in the study. Vivo staff provided study information to their play ambassadors, and those interested in participating contacted the research team. Play ambassadors who had supervised at least one play hub prior to the pandemic were eligible to participate in the study.

Our sample included ten caregivers (nine parents and one grandparent) and four play ambassadors. Caregivers included in our sample self-identified as Chinese (n = 3), Asian (n = 2), or White (n = 5), and their children ranged from ages 3 to 11 years (average = 6.2 yrs; n = 17 children attended the play hubs). Caregivers ranged from ages 33 to 68 years, and eight were female. Play ambassadors included in our sample self-identified as White or Canadian, had an average age of 23.3 years, and prior to the pandemic all had worked as play ambassadors for a minimum of one month during which they facilitated the play hubs. To protect their anonymity, we are unable to provide additional information regarding participant characteristics. We attained oral informed consent and permission to audio-record interviews from study participants at the start of each interview. The University Calgary Conjoint Health Research Ethics Board approved this study and approved use of oral consent (REB20-0074).

### Data collection

Participants underwent semi-structured interviews with the aim of elucidating their perceptions of play. Semi-structured interviews have been established as an appropriate and useful approach for gathering and exploring participant perceptions and opinions [49]. Two members of the research team trained in qualitative methodology (JP and DG) conducted the semi-structured interviews via telephone or videoconference (i.e., Zoom). Interviews lasted up to one hour. Audio-recordings of interviews were transcribed verbatim. All participants received a $25 gift card for their participation. The first interview was conducted on June 30, 2020 and the last interview was conducted on October 19, 2020.

We developed separate interview guides for caregivers and play ambassadors (S1 Table). The context of the two guides however, overlapped in their focus on play. The caregiver interviews included questions about their own and their children’s experiences of attending the play hubs, perceptions about play (including unstructured and risky play), and perspectives about the play ambassadors’ role in facilitating play. The play ambassador interviews included questions about their perceptions of play (including unstructured and risky play), the training received, and their experiences facilitating the play hubs. Relevant academic play [6,12,17,18,21,22,25–30] and play work literature [4,34–36,38,39], as well as input from Vivo administrative staff involved in the development of the play hub program (e.g., feedback from caregivers and play ambassadors) informed the development of the interview guides.

### Data analysis

Data collection and analysis were conducted concurrently allowing for data collection to be informed by the analysis process [47,50,51]. Caregiver interviews were concluded when data collection reached saturation, which was established when the data obtained through interviews no longer added depth, clarity, or detail to the ongoing thematic analysis [50]. The Play Ambassador interviews were concluded when all four play ambassadors who had expressed their interest in participating had been interviewed. The interview transcripts were de-identified and assigned a pseudonym. To support confirmability of the data [52], we offered participants the opportunity to review their transcripts of which five participants obliged. An inductive thematic approach [51,53] was used to explore participant understandings of play, as well as commonalities and differences in perspectives between caregivers and play ambassadors. The thematic analysis involved six steps as outlined by Braun and Clark [53]: familiarization of the data; generating initial codes; looking for initial themes; reviewing and developing themes; defining and naming themes, and; writing the manuscript. We separately analyzed the transcripts of caregivers and play ambassadors, using line-by-line coding to identify initial codes and look for initial themes, then refined these themes before assessing the convergence and divergence of the themes identified within the caregiver and play ambassador interviews.

Initial codes were generated by the two interviewers (JP and DG). The two interviewers engaged in a process of reflexivity throughout the interview process. They recorded detailed notes, reflecting on their positionality and impact of interactions with participant on the data collected [47,54,55]. The interviewers practiced critical, flexible and theoretical thinking when coding the interview transcripts [47,55]. To enhance credibility, the interviewers met regularly to discuss initial codes and themes, addressing any assumptions and refining interpretations [54]. These codes were then collaboratively reviewed by three other team members (JP, DG, and CN), facilitating rigorous discussions and finalizing of themes. Our analytical approach leveraged the diverse expertise of our team, enhancing the credibility, trustworthiness, and depth of our findings [54]. To maintain an audit trail, we documented all analytical decisions as memos [47]. We used NVivo version 12 (QSR International) for the analysis.

## Results

Interviews from caregivers and play ambassadors revealed differences and commonalities in their perceptions of play and the play hubs. We identified four overarching themes: (1) The importance of play; (2) Perceptions of the play environment; (3) Challenges and triumphs in promoting play, and; (4) Play hub impacts on the community.

### Theme 1: The importance of play

Play ambassadors and caregivers described how play is important for child health and development, and provided opportunities for social interaction, enjoyment, exploration, and learning. They spoke specifically to the benefits of children engaging in activities that were unstructured and incorporated some element of risk.

### Opportunities for social interaction

All play ambassadors and caregivers highlighted play as being important for social development. Play ambassadors spoke to the benefits of the social interaction provided through play:

“I think that solo-play has its own benefits 100%, but I think that a lot of the benefits of play come with social interaction, and I think that’s where you reap the greatest rewards.”

(Jordan; play ambassador)

Caregivers also noted the benefits of play for encouraging social interactions and relationship building within families and among others:

“They’re in-fighting at home when they’re bored, but when they are out playing, then they’re strategizing and helping each other. I think that that builds their relationship as brothers.”

(Melissa; caregiver)

Similarly, Daniel (caregiver) described community based play as an opportunity for “being able to play with multiple people, trying to get new friends or old.”

Beyond the forging and strengthening of relationships, caregivers highlighted how the social aspects of play encouraged teamwork and the development of skills that were important for navigating everyday social situations:

“I think it’s very important that children interact when they’re really young because that’s when their brain is slowly growing…where they’re still learning and growing, that is the time, especially motor skills, developmental skills or even just thinking skills, it really helps improve and enhances as they continuously play throughout, and I think that’s how they learn and engage with society if that makes any sense.”

(Lisa; caregiver)

“…you learn a lot about getting along with people… you get to experience getting along with people and learning bigger concepts like cooperation and sharing and stuff that you’re going to need in life.”

(Sandra; caregiver)

Recognizing play as social, and a space for social development was a sentiment shared unanimously among play ambassadors and caregivers, and there were similar convergences in their discussion of play concerning other areas of child development.

### Child health and development

Play ambassadors and caregivers alike discussed the importance of play in supporting healthy child development. The play ambassadors highlighted play as an “intrinsic ability” and essential to children’s lives.

“…play is one of the most inherent and intrinsic abilities that any person has. Everyone knows how to play and play is a defining factor in a lot of kids’ lives as to how they continue to develop growing up in life as well.”

(Morgan; play ambassador)

Echoing this perspective, caregivers discussed the physical and mental health benefits associated with play, and the importance of play for the development of new skills:

“They get outside. They’re generally much happier. They get to expend their energy. I think that it helps them sleep better at night…”

(Jessica; caregiver)

“I would say for the kids, the play is actually experiencing different stuff, stretching their body, learning different texture of different stuff and then how to use the imagination to play with the same stuff but with different methods, something like that, just experience for them.”

(Eric; caregiver)

In defining play, caregivers often highlighted those aspects of play that are unstructured:

“I guess play means to me is unstructured, it’s very spontaneous. It can be imaginative, using your imagination. It can be by yourself. It can be with other kids or other adults even, and I think it’s… the spontaneous nature of playing, I don’t know… messing around and having fun.”

(Heather; caregiver)

“Freely chosen, child-directed, based on their own experiences.”

(Meg; caregiver)

Most caregivers spoke to these “unstructured” or “child-directed” and “freely-chosen’ aspects of play, especially when discussing how play can facilitate exploration, creativity, and enjoyment:

“It is anything that’s fun, and exciting and something that you want to do, rather than [you’re] forced to go do. Play is, “I get to play.” Play is learning, and play is experiencing and growing. It’s a very all-encompassing word.”

(Melissa; caregiver).

While many caregivers spoke primarily to the benefits of unstructured (i.e., self-directed) play, others spoke to the benefits of structured or organized play:

“…playing means going out and experiencing new things, and growing, and testing yourself, but it also means sports. It also has that connotation of the rules and playing in a team…play helps kids learn. It helps them see what they’re capable of. It helps them build relationships with other people.”

(Melissa; caregiver)

Unlike caregivers, play ambassadors mentioned only the beneficial aspects of unstructured play but not structured play. Nevertheless, play ambassadors and caregivers alike recognized the intrinsic value of play generally, especially in supporting children’s learning and growth.

#### Benefits of risky play

Play ambassadors and caregivers shared perspectives of play extended to the importance of encouraging risky play. They considered risky play necessary for children to identify their physical boundaries, to assess risky situations, to develop confidence, and to develop problem-solving capabilities not just in the playground, but also in everyday life:

“As kids continue to grow up, a large part of their play experience is what develops their ability to do a risk/benefit assessment of their own.”

(Morgan; play ambassador)

“Risk, it’s a positive… Risks are what push boundaries. Risks are what develop what we’re trying to develop in these kids.”

(Jordan; play ambassador)

Similarly, caregivers described how risky play offered children opportunities to make and learn from mistakes to develop skills needed for adulthood:

“It’s [a] time for [kids] to try to learn and basically know that if they make a mistake or if something happens that they can get back up, they can try a different way or not do that again.”

(Daniel; caregiver)

“It works on their judgment skills. As an adult, we’ve gone through that experience and had a chance to rationalize and think about it and use our judgment. Kids need those experiences.”

(Kim; caregiver)

Caregivers also noted that prohibiting children from participating in risky activities could be detrimental, as it can invoke fear and negatively affect physical literacy:

“One thing that really comes to mind is that philosophy of risk-taking and allowing kids to take risks. I read a lot of the ParticipACTION Reports that come out, and they talk about the things that our kids are missing right now… dodging because dodgeball is not allowed in schools anymore or taking risks because parents are afraid that if their kids climb to the top of the playground, they’re going to fall and get hurt. We’re taking the risk of getting hurt so far that we’re not allowing kids to take the risks that they are able to take, and so kind of living in that fear.”

(Melissa; caregiver)

Most caregivers associated risky play with the potential for physical harm. Caregivers were comfortable with risky play but highlighted the need to balance risk and safety:

“I do like them to take some risks because that’s how they learn, right, that if they fall, they need to learn how to get back up. So, I do like that, so that they’re not always protected and not being exposed, you know what I’m saying, but at the same time, you don’t want them to get hurt too badly. So, I think in general, I am willing to let them take risks if it’s age appropriate.”

(Lisa; caregiver)

“I think a certain level of risk is important to push you, to gain confidence, to expand your skill set and your knowledge base and so forth. I think I wouldn’t want him doing anything super dangerous, but moderate risk is pretty important, I think to push beyond your comfort zone.”

(Sandra; caregiver)

Play ambassadors shared similar sentiments about balancing risk and safety when encouraging risky play, and described strategies for navigating this space:

“My favorite thing to tell kids was that we don’t say no. I tried to make a point of not ever telling a kid no… as long as you’re going to have fun, as long as you’re going to keep yourself and others safe, I’m not going to tell you no.”

(Morgan; play ambassador)

This balancing between risk and safety was often discussed in reference to specific play objects and environments, with play ambassadors and caregivers both reflecting on how children’s access to different play spaces and play materials impacted their ability to play freely and take risks.

### Theme 2: Perceptions of the play environment

Play ambassadors and caregivers described their experiences of the play hub environment. Notably, they shared their perspectives about how the different loose parts available at the play hubs, shaped children’s play.

#### Loose parts for promoting play

Play ambassadors and caregivers explained how the loose parts encouraged different and diverse types of play. Reflecting on their experiences of supervising the play hubs, play ambassadors highlighted how there were observable patterns in how children engaged with the loose parts:

“There’s a lot of activities where they’re very drawn to because they’re familiar with it…fort-building is always a huge popular one, when you have blankets and all that stuff laid out. We have a bunch of kitchen supplies, like pots, pans, spoons, that sort of thing and that’s always super popular, the pretend play aspect of it. Like they make mud soups, mud pies, all that… It’s really interesting how even though there is kind of a pattern, the way of how all the kids play. They take it in a very different direction.”

(Sam; play ambassador)

Morgan (play ambassador) mentioned that children used traditional materials available in the play hubs (e.g., sports equipment) in a predictable way (e.g., physical play):

“If there were balls in the space, kids would resort to playing soccer, kicking the ball, throwing it, and playing volleyball.”

Jordan (play ambassador) explained that non-traditional materials available in the play hubs often engaged children in exploratory and imaginative play:

“A lot of [play at the events] was exploratory. A lot of them have never been able to do stuff like that and bring out VCRs and DVDs stuff like that, and hammers and screwdrivers and stuff like that nearby. So, they were able to smash things, take things apart, see how they work on the inside… then there was some imaginative light…They’d look at something and they’d think of something and then there’d be that creative, imaginative solo or social play in that aspect.”

Caregivers attending the play hubs with their children also observed that different materials facilitated certain types of play:

“…being able to go and climb on c-cans and throw tires around… My boys are busy and active, and that was a completely different experience because it wasn’t traditional. It was totally out of the box… Whereas the ones [play hubs] at the actual playground, they were more equipment-based like frisbees or building blocks or games…”

(Melissa; caregiver)

Additionally, when reflecting on the play hubs and different play environments, play ambassadors and caregivers often noted their appreciation of being able to utilize outdoor spaces.

#### Benefits of outdoor play

Play ambassadors and caregivers recognized that space, location, and park infrastructure influenced how children play. Play ambassadors observed that fixed-manufactured park infrastructure and natural elements, such as trees and hills, facilitated play differently:

“A lot of our sites do have playgrounds; the playground equipment would get utilized more. For example, like if we had built or created another swing, or we’re going to use one of our tunnels that we bring to slide down the actual slide. So, [children] definitely utilize the playground equipment more.”

(Sam; play ambassador)

Similar to playgrounds, play ambassadors noted that children used natural park features incorporated into the play hub spaces for exploratory or imaginative play:

“There’s tons of natural spaces for kids to hide… There’s trees, there’s hills, there’s ways to engage with water play and the wind… and that’s a part of play too, engaging with the elements of nature to play.”

(Morgan; play ambassador)

Caregivers perceived that their children enjoyed playing at the play hubs, noting that the outdoor environment offered them freedom to move around and explore. Some caregivers had attended both indoor and outdoor play hubs hosted by Vivo and noted differences in their children’s play:

“Whereas the outdoor one seemed more free-flowing and much more, well there’s more room of course… more space… So, I think it’s kind of freer that way for the kids to explore.”

(Sandra; caregiver)

While in many ways the play hubs mitigated one barrier to children engaging in unstructured play, play ambassadors and caregivers highlighted other challenges associated with facilitating this type of play intervention.

### Theme 3: Challenges and triumphs in promoting play

Play ambassadors reflected on their experiences of facilitating play during the play hubs, sharing stories about their challenges and successes. Caregivers offered details about their family’s experiences of the play hubs and their interactions with the play ambassadors.

#### Letting go of control and encouraging unstructured play

Play ambassadors noted that facilitating unstructured play required them to be more open to changes in their ideas about play and caregivers reflected on how the lack of organized activities at the play hubs encouraged unstructured play. Play ambassadors shared how they had to shift some of their own perspectives, or remind themselves of their role:

“I’m definitely kind of a control freak. It’s helped me let go of that control, not being so worried about things not going one way. Kids are going to take it in a million different directions, whichever direction it takes… go with the flow.”

(Sam; play ambassador)

“We were always worried about where we were stepping too much into a kid’s creativity, and when we were no longer enforcing child-led play… you want imagination to come and start from the children themselves. So, we were always worried if something that we’re doing was impeding or stepping on their creativity. And it was hard to balance the, "Am I doing this to encourage their imagination? Or am I now enforcing my own adult thinking on them?"

(Cal; play ambassador)

Play ambassadors also reflected on the challenge of translating this hands-off approach to caregivers, as caregivers at times wanted to interfere in their children’s play.

“I would say some parents would kind of direct the child’s play. They would bring them like a shovel and be like, “Build a snowman.” And the kid would be like, “Okay.”

(Sam; play ambassador).

“[If] parents felt like their kids weren’t doing enough, parents, who are maybe more used to a structured environment, would encourage their kids to do something else, as opposed to what their child was previously doing… The word that we use is ‘adulterate’ the play space.”

(Morgan, play ambassador).

Some caregivers reflected on the “lack of structure” of the play hubs, highlighting the disconnect between the goals of the play hub, and their expectation for their children’s play activities:

“They had all the different toys and equipment in certain areas where they allowed the kids to just play. But they could try and get a little bit more… organized… set up a small game or something like that. Where they could switch in and out kids to play or go like a one-on-one type situation with that.”

(Daniel; caregiver).

Although some caregivers did not understand the unstructured nature of the play hubs, caregivers who did, shared enthusiastic appreciation for this type of play.

“Having that opportunity that says, "Yes. Yes, absolutely, go climb. Do whatever. You want to try to hook this rope up to the top of the c-can, and then down, and then try to zip line, yeah. Go for it. Let’s see if we can make it work." But having that done in an environment that let’s test our theories before we get hurt. That was probably their favorite part.”

(Melissa; caregiver).

Encouraging unstructured play offered a unique challenge for play ambassadors in that they were navigating not only the difficulty of their own interference in children’s play, but also the expectations of caregivers attending the play hubs.

#### Navigating caregiver expectations

Play ambassadors described challenges when managing caregiver expectations of play. Challenges arose from caregiver expectations of what play “should” look like and how “involved” a play ambassador should be:

“I think a lot of parents see the activity as, ‘Oh, what are you guys really doing?’ There’s not really a lot going on… trying to educate parents on how they can play with their kids without directing the play or without facilitating a structured experience for them or telling them how to play.”

(Morgan; play ambassador).

“‘Okay, parents, let’s step away from our kids for a minute and let’s talk about…’ Then they did some parent education, which I thought was really, really nice and really quite necessary. Then you hear what parents are feeling. You hear what parents are thinking.”

(Melissa; play ambassador).

Despite play ambassador efforts to educate caregivers, some, especially those with younger children, found it challenging to allow their children to play alone. Some caregivers perceived the age differences between children attending the play hubs as a risk:

“We kept on watching him and trying to make sure that he stayed away whenever it seemed like some of the older kids were playing with some of the equipment. We tried to keep him with the younger kids and tried to keep him in certain areas or certain sections.”

(Daniel; caregiver).

“I guess, the only thing is if there’s a wider age gap, like if there’s older kids, that’s the only thing I’m more concerned. If they play hockey together, they might be more rough and bump into each other and hurt [the younger kids] because they’re so much younger… There’s a risk that they would bump into the younger ones.”

(Lisa; caregiver).

However, on occasions play ambassadors had to intervene when they observed caregivers encouraging children to take risks that were outside their comfort level:

“And we would always tell parents to not put their kids up [up on c-cans themselves] because a large portion of risk is also subjective to a kid’s perception. So, if a kid is too uncomfortable, we don’t want to push them so much that it becomes very scary for them, and they don’t ever want to do it again.”

(Morgan; play ambassador).

Although the role of “unstructured play educator” was consistently challenging for play ambassadors, their efforts were noticed by caregivers who reflected on the value of having ambassadors to facilitate this type of play.

#### The role of play ambassadors

Caregivers highlighted that the play ambassadors were knowledgeable about play and were essential to the play hubs, bringing positivity and encouragement to their children’s play:

“They’re so knowledgeable too… I wasn’t aware of all the stuff they were keeping in the c-cans, so she would just bring stuff out based on what our kids were doing. I thought that was good. I think they do have a very valuable role‥ [the play ambassador] brought some ideas to light for us in terms of just ideas on different things to play in and still keeping it that unstructured play.”

(Heather; caregiver).

“I think ambassadors make a big difference because they are more interactive with their kids. I think they may bring more experience, positive experiences, and encourage them to play more.”

(Lisa; caregiver).

Caregivers also appreciated that the play ambassadors gave them “peace of mind”, ensuring their child’s safety while also encouraging them to participate in risky play:

“[they] just ensure that there is group safety, but there is room for individual thought… You can never make it risk-free, but because it’s more natural, they let them follow the natural flow and just observed to make sure that they were safe. But learning to play with some risk is important.”

(Nancy; caregiver).

Play ambassadors shared their enjoyment and pride in supporting unstructured play. They appreciated the opportunity to encourage discovery and exploration and to foster community connections:

“I just like the fact that I get to interact with kids and see where they’re at, and I guess learn new things about the way that they work, and the way that they do things. I feel like my ideas are the preconceived notions about what a kid’s limits are, they’re always challenged that way, and this is just another opportunity to see that.”

(Cal; play ambassador).

“I love working with kids. I think kids are some of the most incredible tiny humans in the whole world. I think that one of the things that inspired me the most about becoming a play ambassador was the idea of being able to facilitate an experience for kids and families…those connections in my community and surrounding area, using play to do that was something I hadn’t thought of before and what I found was so wonderful about all of that was I also think it was an opportunity for me as an adult to have a childhood all over again, in essence.”

(Morgan; play ambassador).

Caregiver appreciation of the play ambassadors and play ambassador’s sentimental reflections about play, speak to the relationships and sense of community that were established through the play hubs.

### Theme 4: Play hub impacts on the community

Play ambassadors and caregivers described the benefits of delivering the play hubs in the community. They also spoke to how the play hubs fostered community engagement.

#### Access to novel opportunities

Caregivers appreciated not only that their children had an opportunity to attend the play hubs, but that they offered a novel opportunity for play:

“I think it’s just a positive experience that’s not the same as everything else we do. And that it’s not in the house on a game, on a TV, on an organized structure. The event is there, but how they do it is kind of up to them.”

(Nancy; caregiver).

“The stuff that is there, I cannot find in other places… not just something you can find in the playground, and the kids [have] never tried something like that. So, it’s a good experience. I can spend some time with the kid to try something new.”

(Eric; caregiver).

The play hubs attracted families, who connected through their appreciation for an opportunity for their children to have new experiences, and to connect with new people.

#### Connecting the community

Play ambassadors and caregivers described how the play hubs fostered social interactions among children and caregivers. Play ambassadors noticed that some families used the play hubs as an opportunity to socialize, and that in some instances, these interactions developed into new friendships. The play ambassadors and the play hubs were conduits for bringing families together:

“Some families make a point of coming with friends, so we’ll put out chairs and stuff and sometimes you’ll have 10 adults just hanging out by the fire discussing and having a good time while their kids play, so it’s almost like a social activity that friends in the neighborhood can go do with each other.”

(Jordan; play ambassador).

Play ambassadors reflected on the popularity of the play hubs and observed repeat visits by the same families.

“Right up till when we closed the project because of COVID, we had a lot of returning families coming again and again, which is really great. They got to know each other. We got to know them. And the kids just… They could get out of the house, get outside, even if it was cold.”

(Sam; play ambassador).

Congruent with these perspectives, caregivers shared how the play hubs offered their families an opportunity for social interaction and provided them with a sense of community.

“It fosters more sense of community and as something organized that encourages people to go out and play.”

(Lisa; caregiver).

“With him being an only child it’s nice to go out and if the other families are there, other kids, he can socialize… it’s nice for him to go out and play with some other kids and have some fun experiences.”

(Sandra; caregiver).

However, some caregivers felt there was the potential for more community engagement but expressed disappointed that more people did not attend the play hubs:

“I think it’s a good opportunity… It’s a great opportunity to get out and meet some people or do something. And a lot of times there aren’t a whole lot of people there. So, I think it’s kind of disappointing sometimes that there’s not more community participation, but ideally it would be a great way to bring a community together.”

(Sandra; caregiver).

## Discussion

Our study explored the perspectives about play among caregivers and play ambassadors exposed to a community park-based unstructured, loose parts, play intervention (‘play hubs’). We identified four overarching themes: *The importance of play; Perceptions of the play environment; Challenges and triumphs in promoting play*, *and; Play hub impacts on the community*. While their experiences of the play hubs differed, caregiver and play ambassadors shared similar beliefs regarding the benefits and value of unstructured and risky play for promoting child health and development. Our results align with qualitative findings elsewhere that suggest a general consensus among caregivers [21,22,28–30] and playworkers [5,26,56] concerning the benefits of play. Importantly, playworkers and caregivers noted how different environments and materials support children’s play in different ways, which aligns with other findings suggesting that these differences in play are noticeable and relevant [9]. Given that unstructured play opportunities are declining [1,7,8], the play hubs offer a novel approach for supporting unstructured play in children.

The play hubs provided loose parts and incorporated fixed and natural features to encourage outdoor unstructured and risky play in an adult-supervised environment. Consistent with previous evidence, caregivers believed that loose parts play, facilitated physical activity [41,57], improved physical literacy [58], and fostered creativity and social engagement [34,41,42]. Caregivers and play ambassadors perceived that children enjoyed this style of unstructured play because it provided them with freedom and choice. Evidence however, suggests that caregivers and children differ in their preferences for play environments [14]. Notably, in our study some caregivers were bewildered by the unstructured play (loose parts) offered during the play hubs, which intentionally lacked structure and adult-based-rules, i.e., minimal adult supervision and child self-directed play. This bewilderment led some caregivers to intervene during the play hubs to implement more structure and regulate their children’s play. In their interviews, some caregivers, even those who had spoken to the value of unstructured play, called for more structure during the play hubs, such as the implementation of organized games. While the adulteration of play has the potential to undermine the aims of unstructured play [34], caregiver involvement in their children’s play activities can also be beneficial especially in supporting children in developing specific skills or when there is need to intervene in response to the child’s needs and abilities [59]. The wanting of structure and organization during play reported by some caregivers in our study likely reflects contemporary socio-cultural expectations regarding the nature of play, which prioritizes structured activities (i.e., sports and games) over child self-determined free-play [11–13,60].

Risky play challenges children, providing them with opportunities to explore their physical limits, improve their self-confidence and physical literacy, and positively affect their physical and social health [4,21,61,62]. However, risk anxiety among caregivers can limit the opportunities for child-directed play. Excessive caregiver restrictions on play leads to ‘surplus safety’, whereby the small gains in safety fail to outweigh the potential developmental benefits gained from participation in risky play [10]. Caregiver assessment of risk limits during play reflect their child’s age, maturity, abilities and competence, and the play environment [18,19], and likely explains variations in perceived risks of the same activity (e.g., climbing a tree) among different caregivers [6]. In our study, some caregivers were anxious about allowing younger children to participate in play hub activities in close proximity of older children. The play hubs offered a safe environment for children (targeting 5–12 year olds), yet these caregiver concerns were not unfounded. Compared with younger children, older children have a greater risk tolerance, take-on more physically challenging activities, and are more likely to participate in activities involving physical contact (e.g., rough play) [63]. Less experienced and younger children may also model the riskier activities performed by more experienced and older children [63]. Notably, no caregivers or play ambassadors in our study reported observing any physical incidents or injuries at the play hubs.

Navigating the space between ‘adulteration’ of play, and providing structure and safety was identified as a challenge by play ambassadors, who commented on their roles as educator, play facilitator, and safety monitor. Play ambassadors monitored children’s activities during the play hubs, making subtle suggestions to encourage play and creativity, and intervening in situations where risk of harm or injury outweighed the potential benefits. Where community-based play programs attract children of different ages and abilities and where activities and movement of children is unpredictable, playworkers may need to implement strategies (e.g., age-specific play areas and activity scaffolding) to facilitate an age inclusive play environment. Any strategy, however, needs to weigh the benefits of mixing age groups in play settings with the potential loss of opportunity for positive modelling and socializing that might result from separating children of different ages or abilities.

Play ambassadors and caregivers believed that the play hubs provided opportunities for social interaction and facilitated a sense of community. This finding is notable given that parents with a reduced sense of community may be more reluctant to allow their children to play outdoors [14]. Implementing unstructured play programs in public spaces, such as parks, could be one strategy for increasing sense of community by providing families an opportunity to interact, forge relationships, and engage with other community members. Building trust and sense of community has the potential to encourage caregivers to allow their children to participate in more independent and unsupervised outdoor play in their local communities [14].

We acknowledge several limitations of our study. The play ambassadors represented a self-selected group of highly motivated and knowledgeable individuals with an interest in facilitating play. Moreover, caregivers who attended the play hubs were likely already supportive of their children’s play. Nevertheless, the perspectives reflected in our sample align with findings elsewhere [5,21,22,26,28–30,56]. We recruited participants amidst pandemic public health restrictions, likely resulting in fewer study volunteers. The pandemic imposed methodological challenges for health research [64,65], and the lower than expected participation in our study may reflect pandemic attributed increases in research fatigue [65] and unwillingness to volunteer for research. Nevertheless, the corroborating perspectives garnered from play ambassadors and caregivers, strengthens our study interpretations and conclusions. As this study overlapped with the ongoing COVID-19 pandemic, there was for most participants a gap between when they had experienced Vivo’s play hubs and when interviews were conducted. While this may have impacted participants’ ability to recall specific details about the play hubs, the focus of our study was on caregiver overall perceptions of play and play in loose parts environments. The semi-structured nature of the interviews and design of the interview guide, as well as the novelty of Vivo’s play program meant that participants were still able to provide nuanced and insightful opinions and ideas about children’s play behaviour, despite the elapsed time between attending the play hubs and interviews. Our study focused on the perspectives of play gatekeepers rather than children themselves. While research suggests children often have a stronger desire for risk-taking than their caregivers perceive [66] and prefer different play types and environments compared to their parents’ preferences [14], it is the caregivers who primarily decide their children’s daily activities, including play.

## Conclusions

Caregivers and play ambassadors valued unstructured and risky play, supporting its promotion while recognizing the need to balance safety and risk. The play hubs not only encouraged unstructured and risky play among children, but also facilitated social interaction and enhanced sense of community among adults attending these events. Community-based unstructured loose parts play interventions warrant further research due to their potential to positively impact child health. As organizations implement these play initiatives, it is essential to investigate their impact and the lived experiences of both the facilitators and participants involved in these programs.

## Supporting information

S1 Table Primary questions asked during semi-structured interviews with caregivers and play ambassadors. (DOCX)
