# Supplementary material for: It is child’s play: Caregiver and playworker perspectives on a community park-based unstructured play program
Source: PLoS One. 2024 Sep 30;19(9):e0311293. doi: 10.1371/journal.pone.0311293 (PMC11441671; doi:10.1371/journal.pone.0311293)
Supplement: S1 Table — (DOCX) [file pone.0311293.s001.docx]

| S1 Table. Primary questions asked during semi-structured interviews with caregivers and play ambassadors | |
| --- | --- |
| **Caregiver Questions** | **Play Ambassador Questions** |
| How would you explain what ‘play’ means? | How would you explain what ‘play’ means? |
| What are some of the benefits of play? | What are some of the benefits of play? |
| How important is to you that your children take risks during play? | How did your role change when leading activities that may involve more risk? |
| What would you describe are the main benefits of the play hubs for your family? | Describe some of the impacts of the play hubs on the families attending. |
| How important is the role of the play ambassador at the play hubs? | Describe your background and any training that has helped prepare you for this role as play ambassador. |
|  | Tell me about your role with leading play activities during the play hubs. |
